# Failure of rapid diagnostic tests in *Plasmodium falciparum* malaria cases among travelers to the UK and Ireland: Identification and characterisation of the parasites

**DOI:** 10.1016/j.ijid.2021.05.008

**Published:** 2021-07

**Authors:** Debbie Nolder, Lindsay Stewart, Julie Tucker, Amy Ibrahim, Adam Gray, Tumena Corrah, Carmel Gallagher, Laurence John, Edel O’Brien, Dinesh Aggarwal, Ernest Diez Benavente, Donelly van Schalkwyk, Gisela Henriques, Nuno Sepúlveda, Susana Campino, Peter Chiodini, Colin Sutherland, Khalid B. Beshir

**Affiliations:** a PHE Malaria Reference Laboratory, London School of Hygiene and Tropical Medicine, Keppel Street, London WC1E 7HT, UK; b Department of Infection Biology, Faculty of Infectious and Tropical Diseases, London School of Hygiene and Tropical Medicine, Keppel Street, London WC1E 7HT, UK; c Department of Infectious Diseases, Northwick Park Hospital, London North West University Healthcare NHS Trust, London HA1 3UJ, UK; d Haematology Lab, University Hospital Limerick, Ireland; e Department of Clinical Parasitology, Hospital for Tropical Diseases, University College London Hospitals NHS Foundation Trust, London, UK; f CEAUL — Centro de Estatística e Aplicações da Universidade de Lisboa, Lisbon, Portugal

**Keywords:** RDT, Malaria, *pfhrp2*, *pfhrp3*, Deletion, Plasmodium

## Abstract

**Objectives:**

Our objective was to systematically investigate false-negative histidine-rich protein 2 rapid diagnostic tests (HRP2-RDT) in imported *Plasmodium falciparum* malaria cases from travelers to the UK and the Republic of Ireland (RoI).

**Methods:**

Five imported malaria cases in travellers returning to the UK and RoI from East Africa were reported to the PHE Malaria Reference Laboratory as negative according to histidine-rich protein (HRP2)-RDT. The cases were systematically investigated using microscopic, RDT, molecular, genomic, and in *in vitro* approaches.

**Results:**

In each case, HRP2-RDT was negative, whereas microscopy confirmed the presence of *P. falciparum*. Further analysis revealed that the genes encoding HRP2 and HRP3 were deleted in three of the five cases. Whole-genome sequencing in one of these isolates confirmed deletions in *P. falciparum* chromosomes 8 and 13. Our study produced evidence that the fourth case, which had high parasitemia at clinical presentation, was a rare example of antigen saturation (‘prozone-like effect’), leading to a false negative in the HRP2-RDT, while the fifth case was due to low parasitemia.

**Conclusions:**

False-negative HRP2-RDT results with *P. falciparum* are concerning. Our findings emphasise the necessity of supporting the interpretation of RDT results with microscopy, in conjunction with clinical observations, and sets out a systematic approach to identifying parasites carrying *pfhrp2* and *pfhrp3* deletions.

## Highlights

• Malaria cases in UK and Ireland travellers can give false-negative HRP-RDT results.
• Histidine-rich protein (HRP2/3) deletions can cause false-negative HRP-RDT results.
• High parasitemia may give false-negative RDT results due to a prozone-like effect.
• False-negative RDT results may also be a result of low parasite density.
• Next-generation sequencing can elaborate the breakpoints of HRP2/3 deletions.

## Introduction

Malaria rapid diagnostic tests (RDTs) are point-of-care immunochromatographic lateral flow assays which detect malaria antigens in blood from patients with suspected malaria. RDTs play a key role in the diagnosis and appropriate timely treatment of malaria throughout malaria-endemic regions (Bruxvoort et al., 2017). Formats detecting *Plasmodium falciparum* histidine-rich proteins (HRP2, HRP3) are the most widely used RDTs in endemic countries. In non-endemic countries, these are typically used for initial diagnostic screening and/or to confirm the findings of blood film microscopy, which is considered the gold-standard test. The availability of reference laboratory confirmatory diagnosis by microscopy or PCR, in addition to RDT, is strongly recommended (Askling et al., 2012, Houze et al., 2013). This is because RDT detection of *P. falciparum* has lower sensitivity than other methods, and the false-negative rate resulting from this is exacerbated by the emergence of *P. falciparum* parasites that do not express both HRP2 and HRP3 antigens (Berhane et al., 2018, Gamboa et al., 2010). This phenomenon has become a malaria public health concern and threatens malaria control and case management efforts in endemic and non-endemic countries alike (Berhane et al., 2018, Beshir et al., 2017, Gamboa et al., 2010, Grignard et al., 2020, Watson et al., 2017).

*P. falciparum* parasites that fail to express the histidine-rich antigens are known to carry deletions of the *pfhrp2* locus on chromosome 8 (gene ID: PF3D7_0831800) and/or the *pfhrp3* locus on chromosome 13 (gene ID: PF3D7_1372200). Parasites with one or both loci deleted have been identified in the Americas, Africa, and some parts of Asia (Thomson et al., 2020). In Peru and Eritrea, HRP2-based RDTs are no longer deployed due to the high prevalence of HRP-deleted parasites (Berhane et al., 2018, Gamboa et al., 2010). While the global prevalence of parasites with the deletion remains low, and appears to be concentrated in certain populations, there is a growing recognition by WHO that these represent a potential threat to rapid diagnosis of falciparum malaria, a cornerstone of global control strategies (Gatton et al., 2017, Thomson et al., 2020).

The exact biological function of HRP2 is still unclear, but it has been linked with hemozoin formation, a process that converts a toxic, free heme into non-toxic, insoluble hemozoin during hemoglobin digestion (Sullivan et al., 1996). HRP2 has also been reported to be involved in cytoadherence to endothelial cells of postcapillary venules by erythrocytes infected with mature parasite stages. In a mouse model of *P. falciparum* infection, HRP2 is reported to be a virulence factor that may contribute to cerebral malaria (Pal et al., 2016). It is unknown whether *pfhrp2*/*3* deletion alters parasite fitness or disease severity *in vivo*. However, the analysis of genetic crosses between different laboratory strains has not revealed any deviation of Mendelian expectations for segregation of *pfhrp2* deletions in the progeny (Sepulveda et al., 2018).

We have previously identified *pfhrp2*/*pfhrp3-*deleted parasites in malaria cases imported to the UK from Ethiopia, Kenya, Tanzania, Somalia, South Sudan, and Sudan (Grignard et al., 2020). In the present study we report clinical, parasitological, molecular, and genomic details of a series of five *P. falciparum-*positive cases presenting with false-negative HRP2-RDT results in the UK and the Republic of Ireland (RoI) between 2019 and 2021. We also report the successful culture adaptation of one *pfhrp2*/*3*-deleted parasite isolate of Sudanese origin.

## Materials and methods

### Detection of malaria parasites in blood samples

Blood samples and fixed blood films from suspected or confirmed malaria patients are submitted by laboratories to the PHE Malaria Reference Laboratory (MRL) at the London School of Hygiene and Tropical Medicine (LSHTM) for confirmatory diagnosis. Films are examined by microscopy, as previously described (Nolder et al., 2013), and molecular analyses performed if microscopy is inconclusive and/or if there is any discrepancy between MRL and the sending laboratory diagnostic test results. RDTs are not used routinely, and are only deployed (according to manufacturer’s instructions) when MRL microscopy results indicate a discrepancy with the sending laboratory’s RDT findings.

Deletions and the presence of genetic polymorphisms located in *pfhrp2* and *pfhrp3* are suspected when *P. falciparum* parasites are identified by microscopy or when *P. falciparum* DNA is confirmed using molecular analysis, and the sending laboratory has made an incorrect diagnosis based on RDT findings alone, such as reporting a non-falciparum malaria infection as the result.

### Molecular detection and speciation

Molecular species diagnosis in the MRL combines nested PCR with species-specific 18S ribosomal gene amplicons identified using gel electrophoresis (adapted from Calderaro et al., 2007, Padley et al., 2003, Snounou et al., 1993), and a probe-based qPCR method that discriminates *Plasmodium* species (adapted from Shokoples et al., 2009).

### Identification of *pfhrp2*/*pfhrp3* deletion mutants

Where *pfhrp2*/*3* deletions/mutations are suspected by the MRL, a panel of RDTs that target either *pfhrp2*/*3* or *Pf*LDH antigen are used to investigate. Available tests include: Carestart™ Malaria Rapydtest® (HRP2/pLDH combo Pf/Pan-genus, Apacor); Binax-NOW Malaria (Pf HRP2/pan-genus aldolase, Alere) and OptiMAL-IT (Pf LDH/pan-genus LDH, Bio-Rad).

DNA was purified by a robotic DNA extraction system (Qiagen) following the manufacturer’s instruction, as previously described (Robinson et al., 2019). Molecular testing for gene deletions was by assessment of parasite genomic DNA using a multiplex qPCR assay, as previously described (Grignard et al., 2020). Briefly, the method uses a multiplex assay targeting *pfhrp2* and *pfhrp3* as well as parasite reference and human house-keeping genes. Details of the genes and components of the qPCR have previously been described (Beshir et al., 2020, Grignard et al., 2020).

### Genomic analysis of *pfhrp2* and *pfhrp3* loci

To confirm the *pfhrp2*/*3* deletion and determine the extent of chromosome deletions, whole-genome sequencing (WGS) was performed using a previously described selected whole genome amplification (sWGA) method (Ibrahim et al., 2020, Oyola et al., 2016). The method selectively amplifies parasite DNA using short oligonucleotide probes of 8–12 mers as primers and enriches parasite DNA to generate whole-genome sequencing data from low-parasitemia samples. Details of the SWAG method, purification of the amplified samples, and library preparation of purified samples have previously been described (Ibrahim et al., 2020). All sequencing reactions were performed using paired (2×) 150 bp reads.

### WGS sequence data analysis and adaptation of parasites to *in vitro* culture

The methods for whole-genome sequence data analysis and adaptation of *P. falciparum* isolates to *in vitro* culture are detailed in the supplementary material.

### Ethical approval

Ethical approval for the samples from MRL patients was obtained from the NHS England Research Ethics Committee (18/LO/0738). Ethical approval for the laboratory work involving MRL samples was obtained from LSHTM Ethical Review Committee (#14710).

## Results

### Patients

#### *Patient 1* (sample S105)

In March 2019, a 72-year-old Sudanese female presented to the emergency department at a hospital in England with a 1-day history of symptoms suggestive of malaria. The patient had recently returned from visiting family in Sudan.

Blood films examined by the sending laboratory suggested malaria parasites which, following testing by Carestart RDT, were found to be positive for LDH antigen (pan-genus) only. The sample was referred to the MRL at LSHTM for confirmation of *P. vivax*.

#### *Patient 2* (sample S160)

In September 2019, a 17-year-old British male of Somali heritage presented to the emergency department at a hospital in London with a 10-day history of symptoms suggestive of malaria, having returned from a 2-year stay in Uganda the day before clinical presentation. The patient had started feeling unwell in Uganda, presented to hospital, and been treated with antibiotics. He reported not receiving antimalarial treatment because his malaria diagnostic test was negative.

A provisional diagnosis of *P. vivax* was made after testing by Binax RDT, which was found to be positive for aldolase antigen (pan-genus) only. However, following microscopy, this diagnosis was revised to severe *P. falciparum* with > 20% parasitemia, and the patient was admitted to the intensive care unit, where a course of appropriate treatment was started. The hospital laboratory performed a Carestart RDT, which, despite the presence of microscopy-confirmed *P. falciparum* hyperparasitemia, generated only a weak positive signal for *Pf*HRP2, but was positive for pan-LDH antigen. Blood films and an aliquot of EDTA blood were referred to the MRL for confirmation of species and investigation of inconsistency in RDT results. Biochemical data indicated that the patient had multiple markers of severe malaria (data not shown).

#### *Patient 3* (sample S194)

In January 2020, a 28-year-old male returned to the UK from a 1-week trip to Ethiopia to visit friends and family. He presented to the emergency department at a hospital in London 3 days after arrival, reporting a 5-day history of symptoms consistent with malaria. Scanty trophozoites of *P. falciparum* were reported despite a negative result using Binax RDT. The sample was sent to the Hospital for Tropical Diseases (HTD) in London for confirmation, because it operates a 24-h out-of-hours malaria diagnosis service. The sample was initially reported as *P. malariae* following on-call microscopy together with a Carestart RDT result negative for the *P. falciparum* HRP2/3 antigens, but subsequent examination of the blood-film by HTD microscopists on the next working day reverted the previous diagnosis to *P. falciparum* infection and suspected false-negative RDT. The sample was sent to the MRL for molecular confirmation.

#### *Patient 4* (sample S204)

In March 2020, a 55-year-old male returned to the RoI following a 3-week trip to Sudan, where he had been visiting friends and family. He reported presenting for medical care in Sudan, with symptoms suggestive of malaria, 6 days prior to his return to RoI. Tests for malaria were reported to be negative. The patient then presented to the emergency department at a hospital in the RoI 3 weeks after arrival, a delay likely to have been caused by the rapidly emerging SARS-CoV-2 pandemic. Malaria parasites were seen on blood films and a Carestart RDT was positive for pLDH (pan-genus) antigen only, indicating a non-falciparum infection.

Samples from the patient were received by the MRL for confirmation; *P. falciparum* was diagnosed by microscopy and confirmed by molecular tests. RDTs were performed, confirming the sending laboratory’s findings that the sample was negative for the HRP2/3 antigens. The sample was received within 72 h of being taken from the patient, and an aliquot was successfully established in long-term culture, designated as the parasite line HL2004.

#### *Patient 5* (sample S227)

In January 2021, a 51-year-old female returned to the UK from 6-week stay in Zambia where she has been visiting family. The patient presented to a hospital in England in February and was diagnosed microscopically with malaria, species unknown, with scant parasites seen. A Carestart RDT was negative. Slides and an EDTA blood sample were sent to the MRL for confirmation and species diagnosis.

*P. falciparum* was diagnosed in the MRL by microscopy: early and late trophozoites with Maurer’s clefts were seen. The sample was tested with Carestart, OptiMAL, and Binax RDTs and all three were negative for *Plasmodium* antigens, while Alethia™ Malaria LAMP (Meridian Bioscience) was positive. The qPCR indicated that the infection below 200 p/μL. An aliquot was successfully established in long-term culture, designated as the parasite line HL2005.

### Diagnostic test results

Diagnostic test results and investigations for the five blood samples are summarized in Table 1, Table 2. In each case, despite all five infections being confirmed as *P. falciparum*, inconsistencies in RDT results reported by the sending laboratory were also found by the MRL. These included weak or negative results obtained for HRP2/3 antigens detection by Carestart or Binax test kits, despite positive results for the optimal *Pf*LDH antigen. We therefore suspected possible deletions of the respective genes in all five cases, and proceeded to assess the genomic status at both the *pfhrp2* and *pfhrp3* loci using multiplex qPCR.

**Table 1:** Summary of investigations for blood samples from five malaria patients referred to the MRL following inconsistent RDT results.

Sample reference	Travel history	Carestart Pf HRP	Binax Pf HRP	Optimal Pf LDH	Carestart Pan-genus LDH	Binax Pan-genus aldolase	Optimal Pan-genus LDH
S105	Sudan	*NEG*	*NEG*	*POS*	*POS*	*POS*	*POS*
S160	Uganda	*WEAK*	*NEG/WEAK*	*POS*	*POS*	*POS*	*POS*
S194	Ethiopia	*NEG*	*NEG*	*POS*	*POS*	*POS*	*POS*
S204	Sudan	*NEG*	*NEG*	*POS*	*POS*	*POS*	*POS*
S227	Zambia	*NEG*	*NEG*	*NEG*	*NEG*	*NEG*	*NEG*

Pf: *P. falciparum-*specific.

LDH: parasite lactate dehydrogenase detectable by specific monoclonal antibodies (MAb) in the RDT.

Pan-genus: where RDT deploys an MAb that detects antigens from all members of the genus *Plasmodium.*

**Table 2:** Microscopy, qPCR and parasite culture results for blood samples from five malaria patients referred to the MRL.

Sample reference	Travel history	Initial diagnosis	Microscopy	*P. falciparum* DNA detected?	Culture established?
S105	Sudan	*P. vivax*	*P. falciparum* trophozoites P ∼ 0.02%	*P. falciparum* DNA detected Ct 20.08	No
S160	Uganda	*P. vivax*/*P. falciparum*	*P. falciparum* trophozoites P ∼ 24.3%	Not done	No
S194	Ethiopia	*P. falciparum*/*P. malariae*	No films received	*P. falciparum* DNA detected Ct 24.71	No
S204	Sudan	Malaria parasites seen; unspecified	*P. falciparum*, all stages P ∼ 0.1%	*P. falciparum* DNA detected Ct 22.88	Yes (HL2004)
S227	Zambia	Malaria parasites seen; unspecified	*P. falciparum*, late trophozoites P ∼ 0.02%	*P. falciparum* DNA detected Ct 25.32	Yes (HL2005)

Ct: cycle threshold — amplification cycle at which threshold for positivity is crossed by fluorescent signal in qPCR assays. Mean Ct values are given for two replicates.

### Confirmation of *pfhrp2*/*3* deletion using multiplex qPCR

**Figure 1:**
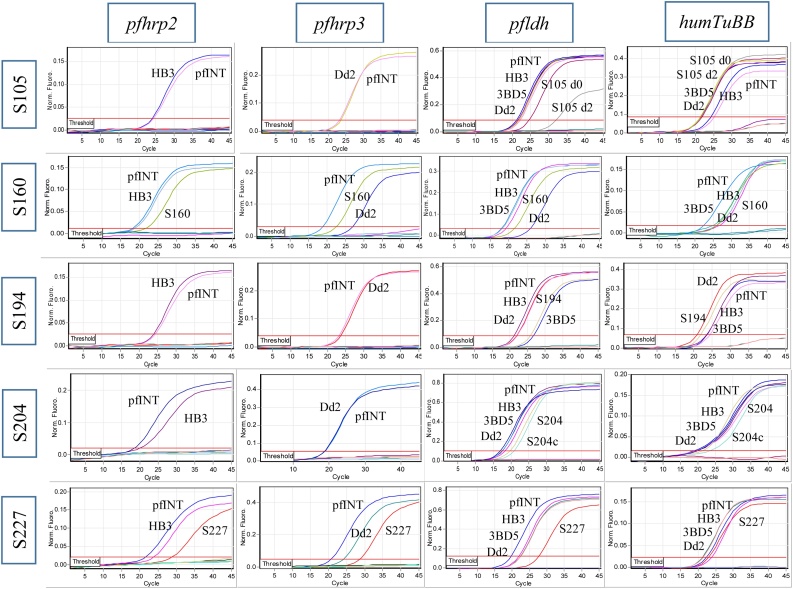
Amplification curves for four different genes in five samples. Three samples (S105, S194, and S204) showed no amplification curves for *pfhrp2* and *pfhrp3* genes, while two samples (S160 and S227) showed amplification curves for both *pfhrp2* and *pfhrp3* genes. All samples showed good amplification curves for both parasite (*pfldh*) and human (*humTuBB*) internal controls. As expected, all the controls (Dd2, INT, 3BD5, and HB3) showed amplification curves or no amplification curves according to their *pfhrp2*/*3* status.

**Figure 2:**
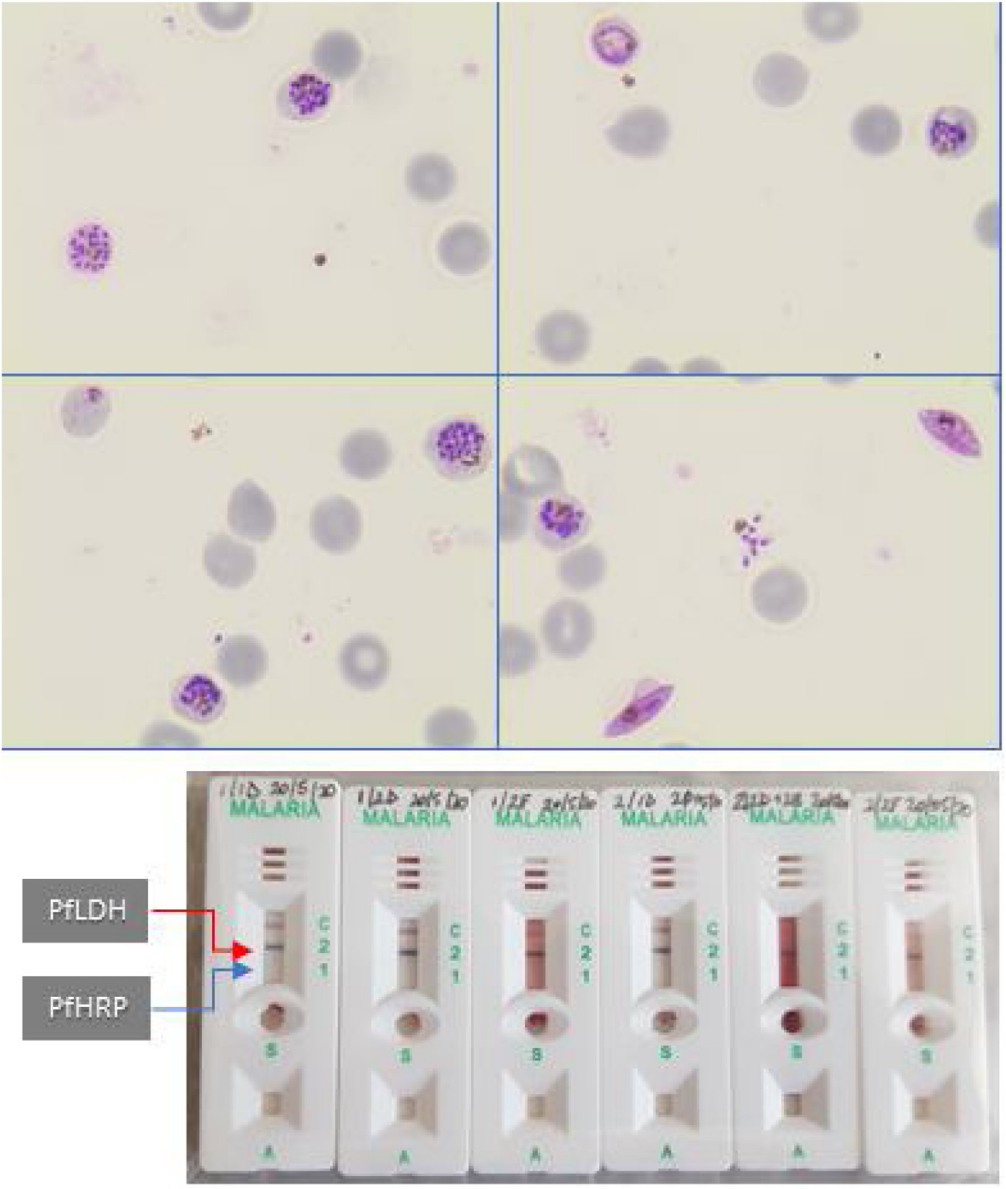
Carestart™ Malaria Rapydtest® detection of LDH but not HRP antigens in six independent culture wells 4 weeks after commencing *in vitro* propagation of *P. falciparum* isolate S204. The upper panel shows four representative Giemsa-stained microscopy fields after magnetic purification, which selectively enriches for late-stage asexual parasites and gametocytes. The lower panel shows readouts from neat culture supernatants from six different culture wells. Following successful cryopreservation, this line was designated HL2004. C, control line; red arrow, expected position of PfLDH antigen detection line; blue arrow, expected position of PfHRP2/3 antigen detection line.

Our one-step quadruplex qPCR assay amplifies three parasite genes (*pfhrp2*, *pfhrp3*, and *pfldh*) and a human gene (*HumTuBB*) in a single reaction. All samples with suspected *pfhrp2*/*3* deletions showed good amplification of the *pfldh* target gene (Figure 1), confirming the presence of good-quality parasite DNA in all samples. Three samples (S105, S160, and S204) were negative for *pfhrp2* and *pfhrp3* genes, as shown by the absence of amplification signal in both gene channels. Surprisingly, two samples (S160 and S227) showed amplification signals with Ct values of 22.0 and 28.6, respectively, for each of the genes, confirming the presence of both *pfhrp2* and *pfhrp3* (Figure 1, second row). The presence of amplification signals for both the parasite and human internal control genes indicated successful DNA extraction and PCR amplification in each case, and positive controls showed the expected amplification signal for all targets. The successfully culture-adapted samples (S204 and s227) were investigated for maintenance of the *pfhrp2*/*3* deletion during the *in vitro* culture adaptation as isolates HL2004 and HL2005. Molecular analysis of the DNA extracted from HL2004 at days 21 and 28 following culture adaptation revealed that the sample generated no amplification signal for the *pfhrp2*/*3* genes (Figure 2), while the HL2005 sample produced a good signal. The reference parasite gene (*pfldh)* produced a good amplification signal for both samples (data not shown). This confirmed that both culture-adapted isolates reflected the results seen in the original patient isolate (Figure 1, row 4).

### Further investigations for S160

Given that qPCR analysis of the parasite DNA from S160 confirmed the presence of intact genes coding for both HRP antigens, sample dilution was performed to test the hypothesis that antigen saturation (a ‘prozone-like effect’) may have accounted for the inconsistent RDT results for HRP antigen detection in this sample. The undiluted blood sample gave inconsistent results with HRP RDT, with a weak positive signal by Carestart™ Malaria Rapydtest® and a negative result by Binax-NOW. The original EDTA sample was diluted 1:10 in uninfected human blood. This dilution tested positive with both RDT formats, confirming the presence of both *Pf*LDH and *Pf*HRP antigens (data not shown).

### Genomic analysis of *pfhrp2* and *pfhrp3* loci and *P. falciparum* isolate culture

**Figure 3:**
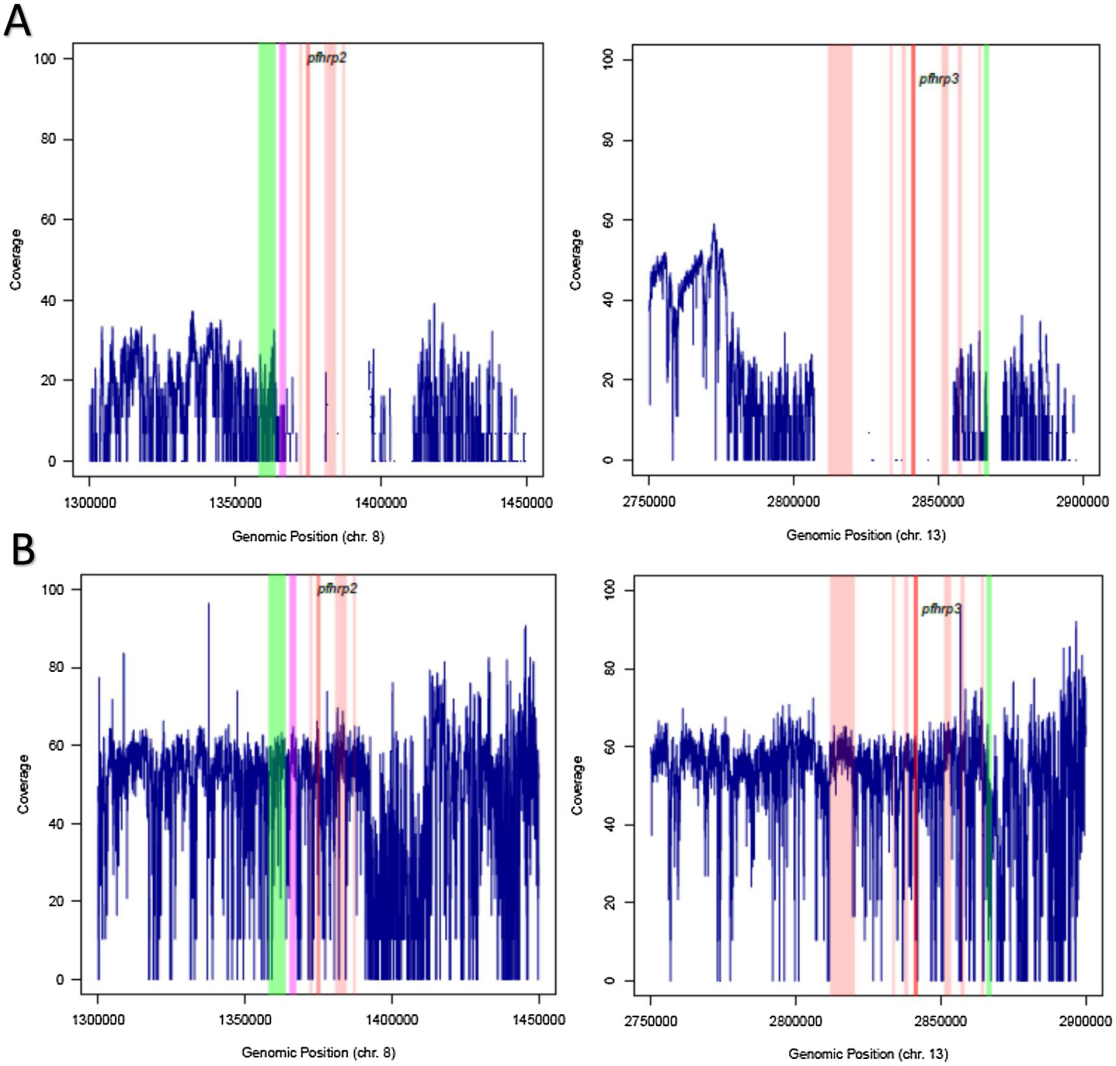
Genomic coverage of *pfhrp2*, *pfhrp3*, and flanking regions for two clinical samples. Short-read sequence coverage is missing around the *pfhrp2* and *pfhrp3* in sample S105 (top panel) but not in sample S160 (bottom panel). (A) *pfhrp2* and *pfhrp3* (dark orange) in clinical sample S105 (top panel) are deleted together with flanking regions that include PHIST, STEVOR, EBL1, RIFIN, PHISTb, and HSP70x (purple) but not PfEMP1 and CLAG8 (green). (B) Both *pfhrp2* and *pfhrp3*, as well as the adjacent genes (colored), show good genomic coverage in clinical sample S160 (bottom panel).

Results for genomic analysis of *pfhrp2*/*3* loci (inculding anlysis about Figure 3) and adaptation of *P. falciparum* isolates are presented in the Supplementary material.

## Discussion

This study presents a systematic investigation of false-negative HRP2-RDT results in travelers to the UK and RoI with imported malaria due to *P. falciparum* infection. Clinical, molecular, genomic, and *in vitro* evidence is provided, confirming the deletion of *pfhrp2*/*3* genes in parasites from three patients with false-negative results by HRP2-RDT. This is the first study to show *pfhrp2*/*3* deletion in patient samples from UK travelers causing false-negative HRP2-RDT results. The study also presents evidence that a false-negative RDT result obtained for a fourth patient was due to a prozone-like effect due to antigen saturation caused by high parasitemia, and for a fifth patient due to low parasitemia.

We have previously identified *pfhrp2*/*3* deletions in a molecular survey of *P. falciparum* parasites imported into the UK (Grignard et al., 2020), but these were not linked to diagnostic test failure. In this study, we show the potentially serious impact of imported parasites with HRP deletions on RDT performance in the UK and RoI. With the increase in frequency of such *P. falciparum* parasites in endemic countries, over-reliance on HRP2-RDT diagnosis in the UK or RoI may delay treatment for sick individuals, with life-threatening consequences. One patient in our series with false-negative RDT results was admitted to intensive care unit with hyperparasitemia; he reported receiving no antimalarial treatment in Uganda prior to travel due to a negative malaria test. Early presentation, rapid diagnosis, and immediate treatment can together greatly reduce severe malaria risk, and are the basis of effective malaria management in non-endemic countries (Checkley et al., 2012).

The prozone effect due to high parasitemia has been previously demonstrated in laboratory studies of both clinical isolates and laboratory cultures (Luchavez et al., 2011). Variation in RDT susceptibility between RDT products was observed, reflecting either the quality of the RDT manufacture or the high specificity of commercially produced proprietary monoclonal antibodies used on the test devices. Other researchers have reported prozone-like effects at parasitemia leves of 5.5–35% (Gillet et al., 2009), and there is evidence that high concentrations of patient antibodies against HRP2/3 antigens may also contribute to the inhibition of RDT recognition of antigens (Ho et al., 2014). Thus *pfhrp2*/*3* deletion is not the only factor that contributes to false HRP2-RDT negative results, and both factors should be considered when faced with HRP2-RDT negative results in patients with suspected malaria. Our findings also emphasize the need for ruling out low parasitemia as a possible factor for false-negative RDT results, as extensively demonstrated in our previous report (Beshir et al., 2017).

For the present study, a multiplex qPCR assay — recently validated as a one-step genotyping method (Grignard et al., 2020) — was used to demonstrate that false-negative RDT results were caused by the deletion of *pfhrp2*/*3* in three patients. Our results confirmed that *pfhrp2*/*3*-deleted parasites are in circulation in Ethiopia and Sudan, as previously reported (Boush et al., 2020, Girma et al., 2019, Golassa et al., 2020, Mussa et al., 2019). Interestingly, a mathematical modelling exercise (Watson et al., 2017) predicted the insurgence of such parasites in African countries with low endemicity, such as Sudan, Somalia, or Ethiopia, which were the travel origins of the patients we analysed. However, it is unclear how extensively these parasites circulate in these countries. At the genomic level, the S105 clinical sample from Sudan exhibited zero sequence coverage at the *pfhrp2* and *pfhrp3* loci, confirming deletion of the two genes and flanking regions. The deletion of a large proportion of loci surrounding *pfhrp2* and *pfhrp3* is similar to the regional loss observed in laboratory lines Dd2 and HB3 (Sepulveda et al., 2018), though the loss in Dd2 is smaller (14 kb) compared with the clinical sample described here (∼50 kb). The implication of the loss of genes adjacent to *pfhrp2* and *pfhrp3* is unknown. It is important to note that the S160 patient had signs and symptoms attributable to severe malaria, and it is not clear whether the presence of HRP2 and flanking regions contributed to the severity (Pal et al., 2016) or it was simply caused by high parasitemia (Kingston et al., 2017). Cytoadherence — a characteristic of cerebral malaria — is thought to be mediated by parasite proteins such as RIFINs, STEVORs, HSP70x, and pfEMP1 (Sherman et al., 2003), all of which are located in the vicinity of the *pfhrp2* or *pfhrp3* loci. Whether the loss of *pfhrp2*/*3* or the flanking regions has any role in parasite fitness or virulence requires thorough investigation in future studies, including precision mapping of chromosome breakpoints in multiple parasites carrying HRP gene deletions.

Attempts to culture-adapt the clinical isolates was successful for one from a traveller returning to the RoI from Sudan, which lacked both HRP genes, and one from a UK traveller returning from Zambia, which harboured both genes. The growth of HRP-negative parasite line HL2004 was poor compared with other patient-derived lines with intact *pfhrp2*/*3* previously cultured in our laboratory (van Schalkwyk et al., 2013). It is not clear whether poor growth was due to a loss of fitness associated with the deletion of *pfhrp2*/*3*, the delay in establishing the culture, or any other technical factors. Disruption of *pfhrp2* in the 3D7 laboratory line has been reported to have no effect on parasite growth (Yang et al., 2020); however, the *pfhrp2* locus alone was disrupted by gene replacement in this study, and so any fitness impact due to loss of flanking genes was not examined. The exact biological function of HRP2 is still unclear, but it has been linked with hemozoin formation in several studies (Schneider and Marletta, 2005, Sullivan et al., 1996). The *pfhrp2* knock-out 3D7 parasite line has been shown to transcriptionally downregulate enzymes required for heme metabolism in the food vacuole, but to upregulate transcripts for proteins required for heme biosynthesis in the apicoplast, potentially maintaining a relatively unchanged heme level in the parasite (Yang et al., 2020). This might indicate that *pfhrp2* deletion can impact parasite survival in the mosquito and liver stages, as *de novo* heme biosynthesis in the apicoplast has been reported to be essential in the mosquito (Ke et al., 2014) and liver stages (Nagaraj et al., 2013). Clonal expansion of *pfhrp2*-deleted parasites has been observed in Eritrea and Ethiopia (Berhane et al., 2018, Girma et al., 2019, Golassa et al., 2020), but it remains unclear whether the parasites with *pfhrp2* deletion have a fitness advantage or deficit compared with the wild type. In areas where RDT-testing and treatment is a policy, the *pfhrp2*-deleted parasites would evade HRP2-RDT detection and would therefore not be exposed to antimalarial onslaught. This potentially gives *pfhrp2*-deleted parasites survival and expansion advantages over the wild type (Watson et al., 2017). Mosquito transmission studies are now needed to understand the role of *pfhrp2*/3 and flanking regions in the expansion of *pfhrp2/3*-deleted parasite populations in malaria-endemic countries. The gametocyte-producing property of HL2004 suggests that this isolate is ideal for such a study.

Our study had some limitations, including the small number of clinical samples in which the impact of *pfhrp2*/*3* deletion on RDT performance in the UK and RoI was evaluated. Similarly, the precise role of high parasitemia on HRP2-RDT performance, which dramatically affected diagnostic results for one patient reported here, requires assessment in a larger patient series. Finally, the culture-adapted HRP-negative isolate will undoubtedly prove a useful research tool as well as a potential source of reference material for evaluating RDT performance against gene-deleted parasites, but may not provide a generalizable model of the fitness cost of *pfhrp2*/*3* deletion. Isolation of additional culture-adapted HRP-deleted *P. falciparum* lines of clinical origin should be prioritised.

Our investigations enhance the existing evidence base, and support the continued use of RDT as a rapid and convenient screening tool for imported malaria, but one that should only be deployed when backed-up by high-quality microscopy and capacity for DNA-based molecular tests. Although RDT diagnostic failures remain rare among imported UK malaria cases, molecular surveillance of *pfhrp2*/*3* needs to be maintained so that the impact of imported *P. falciparum* with *pfhrp2*/*3* deletion on RDT performance in the UK can be continuously monitored.

## Funding

This work was supported by a Wellcome Institutional Strategic Support Fund granted to KBB (204928/Z/16/Z). DN is funded by the Public Health England Malaria Reference Laboratory. The Malaria Reference Laboratory is funded by 10.13039/501100002141Public Health England (research contract awarded to CJS). The funders did not have any role in study design, data collection, data analysis, interpretation, or writing of the report.

## Conflicts of interest

KBB declares financial support from the 10.13039/100004423World Health Organization. Other authors declare no conflicts of interest.
