# Supplementary material for: Failure of rapid diagnostic tests in Plasmodium falciparum malaria cases among travelers to the UK and Ireland: Identification and characterisation of the parasites
Source: Int J Infect Dis. 2021 Jul;108:137–44. doi: 10.1016/j.ijid.2021.05.008 (PMC8295040; doi:10.1016/j.ijid.2021.05.008)
Supplement: Supplementary file 1 [file mmc1.docx]

**Failure of rapid diagnostic tests in Plasmodium falciparum malaria cases among travelers to the UK and Ireland: Identification and characterisation of the parasites**

Debbie Nolder^1^, Lindsay Stewart^1,2^, Julie Tucker^1^, Amy Ibrahim^2^, Adam Gray^3^, Tumena Corrah^3^, Carmel Gallagher^3^, Laurence John^3^, Edel O’Brien^4^, Dinesh Aggarwal^5^*,* Ernest Diez Benavente^2^, Donelly van Schalkwyk^1,2^, Gisela Henriques^2^, Nuno Sepúlveda^2,6^, Susana Campino^2^, Peter Chiodini^1,5^, Colin Sutherland^1,2^, Khalid B Beshir^2^*

^1^PHE Malaria Reference Laboratory, London School of Hygiene and Tropical Medicine, Keppel Street, London WC1E 7HT, UK

^2^Department of Infection Biology, Faculty of Infectious and Tropical Diseases, London School of Hygiene and Tropical Medicine, Keppel Street, London WC1E 7HT, UK

^3^Department of Infectious Diseases, Northwick Park Hospital, London North West University Healthcare NHS Trust, London HA1 3UJ, UK

^4^Haematology Lab, University Hospital Limerick, Ireland

^5^Department of Clinical Parasitology, Hospital for Tropical Diseases, University College London Hospitals NHS Foundation Trust, London, UK

^6^CEAUL — Centro de Estatística e Aplicações da Universidade de Lisboa, Lisbon, Portugal

Key words: RDT, malaria, *pfhrp2*, *pfhrp3*, deletion, plasmodium

Running title: False-negative malaria RDT results

*Corresponding author:

Dr Khalid B. Beshir

Department of Infection Biology

Faculty of Infectious and Tropical Diseases

London School of Hygiene and Tropical Medicine

Keppel Street

London WC1E 7HT

UK

[khalid.beshir@lshtm.ac.uk](mailto:khalid.beshir@lshtm.ac.uk)

**Supplementary material**

**MATERIALS AND METHODS**

**Genomic analysis of *pfhrp2* and *pfhrp3* loci**

To confirm *pfhrp2*/*3* deletion and determine the extent of chromosome deletions, whole-genome sequencing (WGS) was performed using a previously described selected whole-genome amplification (sWGA) method (Ibrahim et al., 2020; Oyola et al., 2016). The method selectively amplifies parasite DNA using short oligonucleotide probes of 8–12 mers as primers, and enriches parasite DNA to generate whole-genome sequencing data from low-parasitemia samples. Details of the SWAG method, purification of the amplified samples, and library preparation of purified samples have been previously described (Ibrahim et al., 2020). All sequencing reaction were performed using paired (2×) 150 bp reads.

**WGS sequence data analysis**

Sequence data from each sample were subjected to the trimming of raw sequence files using Trimmomatic software (Bolger et al., 2014) and mapping sequence reads to the *P. falciparum* 3D7 reference genome (PlasmoDB) using BWA, as described previously (Ibrahim et al., 2020; Turkiewicz et al., 2020). The genome-wide and *pfhrp2* and *pfhrp3* (*pfhrp2*/*3*) coverage was determined using SAMtools, as described previously (Sepulveda et al., 2018). Detection of *hrp2* and *hrp3* deletion was carried out using similar assumptions and methods to those described previously (Sepulveda et al., 2018). The coverage analysis — the number of reads mapped onto each position — focused on the 100-kb extended loci of *hrp2* (from 1 300 000 to 1 450 000 at chromosome 8) and *hrp3* (2 750 000 to 2 900 000 at chromosome 13).

**WGS data availability**

All raw sequence data, anonymized to remove patient identifiers, were submitted to the European Nucleotide Archive (study accession number TBA).

**Adaptation of *P. falciparum* isolates to *in vitro* culture**

Parasites received at the MRL in EDTA were placed into culture using standard *in vitro* adaptation methods, as previously described, with some modification (van Schalkwyk et al., 2013). Briefly, initial culture conditions for adaptation consisted of RPMI 1640 supplemented with 0.5% (w/v) Albumax II, 10% equine serum, and 2 mM Glutamax^TM^ (Gibco product 35050-038), at 5% hematocrit. Cultures were incubated in an atmosphere of 5% O_2_, 5% CO_2_, 90% N_2_ in a T25 flask, shaking constantly at 60 rpm. On day 10 the medium was modified to RPMI 1640 supplemented with 0.5% Albumax II, 2% AB human serum, and 2 mM L-glutamine. The hematocrit was adjusted to 2% and the cultures transferred to a six-well plate (CytoOne® tissue culture plate). The plate was gassed and shaken at 60 rpm.

**RESULTS**

**Genomic analysis of *hrp2* and *hrp3* loci**

The genomes of two samples (S105 and S160) were successfully sequenced. Read coverage of *pfhrp2* and *pfhrp3* was effectively zero for sample S105 but with normal coverage for sample S160. In sample S105, there was evidence for a 50 kb regional loss spanning the positions of 1 363 000 and 1 413 000 on chr 8 and 2 810 000 and 2 858 000 on chr13, where *hrp2* and *hrp3* are located, respectively (Figure 2). Among the deleted regions flanking *pfhrp2* were loci encoding exported proteins PHISTa (3D7-083175), PHIST (PF3D7-0831900), STEVOR (PF3D&-083200), and RIFIN (PF3D7-0832100), all members of previously described subtelomeric multigene families. The heat shock protein (HSP70x, PF3D7_0831700), a gene believed to be involved in the manifestation of severe malaria, lies very close to the upstream breakpoint and was partially deleted. Two other adhesion-related genes on the same locus — cytoadherence-linked asexual protein (CLAG8, PF3D7_0831600) and a *var* locus encoding an isoform of erythrocyte membrane protein 1 (PfEMP1, PF3D7_0833500) — were both present (Figure 3). Deleted genes adjacent to *pfhrp3* include five genes encoding proteins with export signals (PF3D7_01371700, PF3D7_01371900, PF3D7_01372000, PF3D7_01372100, PF3D7_01372300), acyl-CoA synthase (PF3D7_01372400), and one STEVOR isoform (PF3D7_01372500). Analysis of the genomic data for sample S160 showed sufficient coverage for the extended *pfhrp2* and *pfhrp3* loci on their respective chromosomes, confirming that neither *pfhrp2* nor *pfhrp3* loci were deleted.

**Parasite culture from S204**

The successful establishment of sample S204 in culture, as parasite line HL2004, permitted more detailed analysis of these parasites. Parasite growth was poor in comparison with other patient isolates cultured in our laboratory (van Schalkwyk et al., 2013), and it took some weeks to establish and expand sufficient material to cryopreserve stabilates of the line. On day 20 (D20), when the parasitemia was approximately 0.7%, 5 ml blood was tested using the Carestart™ Malaria Rapydtest^®^ to confirm absence of HRP2. This process was carried out periodically throughout the 34-day continuous culture period. Frozen stocks were made on D14, D17, D20, D24, D26, D32, and D34. Material for DNA extraction was collected on D26 and D34.

At each opportunity to do so, the culture supernatant was tested against RDT for HRP and LDH antigen detection, and in each case was negative for HRP but strongly positive for LDH (Figure 2). Similarly, qPCR testing on D26 and D34 confirmed deletion of both the *pfhrp2* and *pfnrp3* loci at these timepoints (data not shown).

**REFERENCES**

Bolger AM, Lohse M, Usadel B. Trimmomatic: a flexible trimmer for Illumina sequence data. Bioinformatics 2014;30(15):2114–20.

Ibrahim A, Diez Benavente E, Nolder D, Proux S, Higgins M, Muwanguzi J, et al. Selective whole genome amplification of Plasmodium malariae DNA from clinical samples reveals insights into population structure. Sci Rep 2020;10(1):10832.

Oyola SO, Ariani CV, Hamilton WL, Kekre M, Amenga-Etego LN, Ghansah A, et al. Whole genome sequencing of Plasmodium falciparum from dried blood spots using selective whole genome amplification. Malar J 2016;15(1):597.

Sepulveda N, Phelan J, Diez-Benavente E, Campino S, Clark TG, Hopkins H, et al. Global analysis of Plasmodium falciparum histidine-rich protein-2 (pfhrp2) and pfhrp3 gene deletions using whole-genome sequencing data and meta-analysis. Infect Genet Evol 2018;62:211–9.

Turkiewicz A, Manko E, Sutherland CJ, Diez Benavente E, Campino S, Clark TG. Genetic diversity of the Plasmodium falciparum GTP-cyclohydrolase 1, dihydrofolate reductase and dihydropteroate synthetase genes reveals new insights into sulfadoxine-pyrimethamine antimalarial drug resistance. PLoS Genet 2020;16(12):e1009268.

van Schalkwyk DA, Burrow R, Henriques G, Gadalla NB, Beshir KB, Hasford C, et al. Culture-adapted Plasmodium falciparum isolates from UK travellers: in vitro drug sensitivity, clonality and drug resistance markers. Malar J 2013;12:320.
